# Crystallization Tendency of Pharmaceutical Glasses: Relevance to Compound Properties, Impact of Formulation Process, and Implications for Design of Amorphous Solid Dispersions

**DOI:** 10.3390/pharmaceutics11050202

**Published:** 2019-05-01

**Authors:** Kohsaku Kawakami

**Affiliations:** World Premier International Research Center for Materials Nanoarchitectonics (WPI-MANA), National Institute for Materials Science, 1-1 Namiki, Tsukuba, Ibaraki 305-0044, Japan; kawakami.kohsaku@nims.go.jp; Tel.: +81-29-860-4424

**Keywords:** pharmaceutical glass, crystallization tendency, crystallization, nucleation, milling, accelerated stability test

## Abstract

Amorphous solid dispersions (ASDs) are important formulation strategies for improving the dissolution process and oral bioavailability of poorly soluble drugs. Physical stability of a candidate drug must be clearly understood to design ASDs with superior properties. The crystallization tendency of small organics is frequently estimated by applying rapid cooling or a cooling/reheating cycle to their melt using differential scanning calorimetry. The crystallization tendency determined in this way does not directly correlate with the physical stability during isothermal storage, which is of great interest to pharmaceutical researchers. Nevertheless, it provides important insights into strategy for the formulation design and the crystallization mechanism of the drug molecules. The initiation time for isothermal crystallization can be explained using the ratio of the glass transition and storage temperatures (*T_g_*/*T*). Although some formulation processes such as milling and compaction can enhance nucleation, the *T_g_*/*T* ratio still works for roughly predicting the crystallization behavior. Thus, design of accelerated physical stability test may be possible for ASDs. The crystallization tendency during the formulation process and the supersaturation ability of ASDs may also be related to the crystallization tendency determined by thermal analysis. In this review, the assessment of the crystallization tendency of pharmaceutical glasses and its relevance to developmental studies of ASDs are discussed.

## 1. Introduction

Amorphous solid dispersions (ASDs) are among of the most effective enabling formulations for improving the dissolution process and therefore the oral absorption of poorly soluble drugs [1,2,3,4,5,6,7]. Because of their high energy, amorphous solids can reach a supersaturated state during their dissolution process. Although solubilization techniques that increase the equilibrium solubility, including the use of micelles and organic solvents, can inhibit membrane permeation [8,9], it does not happen for supersaturated systems originated from ASDs [10]. It is now widely recognized that the supersaturation created by ASDs can cause phase separation into concentrated and diluted phases, based on the spinodal decomposition mechanism, followed by the formation of a quasi-equilibrium colloidal structure consisting of a concentrated dispersed phase suspended in a diluted continuum phase [11,12]. Although the role of the dispersed phase in the oral absorption is still under debate, this process can maintain high levels of supersaturation for the continuum phase, which are beneficial for oral absorption [13]. The stability of the colloidal phase is significantly influenced by the polymer species [13,14,15]. Since the supersaturation behavior of ASDs, including phase separation and its impact on membrane transport and oral absorption, are outside the scope of this review, readers interested in these aspects are referred to recent studies [11,13,16,17,18] for further details.

Drug molecules in ASDs must remain in the amorphous state to exert their beneficial effects during the dissolution process. Even a trace amount of crystals would undermine these favorable effects, because it induces crystallization after suspension of the ASD in aqueous media [19,20]. Polymeric excipients in ASDs serve not only for improving the supersaturation behavior as mentioned above, but also for inhibiting crystallization of the drug. Miscibility is an important factor for exploiting the stabilization effect by the polymer [21,22,23]. Obviously, the crystallization tendency of the drug molecule itself is another important factor affecting the storage stability.

**Figure 1:**
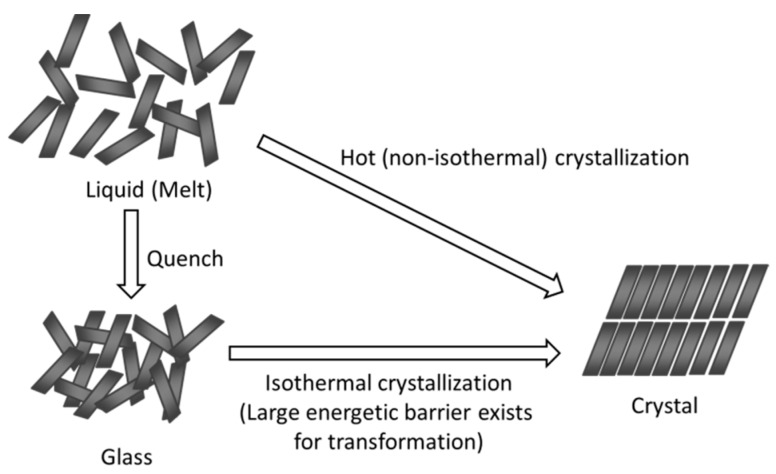
Schematic representation of hot (non-isothermal) and isothermal crystallization.

Table 1 summarizes generally accepted ideas for good glass formers in the case of small organic compounds. Good glass formers tend to have a large molecular weight [24]; other chemical-structural properties of these compounds include a low number of benzene rings, a high degree of molecular asymmetry, as well as large numbers of rotatable bonds, branched carbon skeletons, and electronegative atoms [25,26,27]. Specific tendencies can be found for the physicochemical properties as well. Good glass formers should have a high melting temperature and enthalpy/entropy, as well as a large free energy difference between crystalline and amorphous states [26]. Fragility [28,29], which quantifies the degree of non-Arrhenius behavior of a glass, is another parameter that can correlate with the crystallization tendency [26,30,31]. However, it should be emphasized that the crystallization tendency of a certain compound is frequently determined by observing its crystallization during rapid cooling or cooling/reheating cycles using differential scanning calorimetry, which does not necessarily reflect easiness of the isothermal crystallization, which is of interest for pharmaceutical researchers. The difference between hot (non-isothermal) and isothermal crystallization is schematically illustrated in Figure 1. Hot crystallization proceeds upon a decrease in free volume, and each molecule has a relatively high conformational flexibility during the crystallization. On the other hand, isothermal crystallization occurs under almost constant volume, and the molecular motion is more restricted. Crystallization can only be achieved after overcoming the energetic barrier to structural transformation, in which noncovalent “weak” interactions play an important role, unlike in inorganic glasses.

The following sections review the crystallization tendency of pharmaceutical glasses, with emphasis on relationship with their chemical structure, remark on its evaluation process, relevance for glass properties including the storage stability (i.e., isothermal crystallization), relevance to manufacture, and possible correlation with the supersaturation ability. In addition to discussion on ideal glasses that can be prepared by melt–quench procedure, the stability of real glasses, which are prepared through formulation process such as milling, is also discussed.

## 2. Classification of Crystallization Tendencies

In the field of pharmaceutical sciences, many research groups have evaluated the crystallization tendency of drug molecules by applying a cooling/reheating cycle to the melt in a differential scanning calorimetry (DSC) [26,32]. The following classification, as proposed by Taylor et al. [26], is widely recognized:

Class 1: Compounds that crystallize during cooling from the melt at 20 °C/min.
Class 2: Compounds that do not crystallize during cooling from the melt, but crystallize during subsequent reheating at 10 °C/min.
Class 3: Compounds that do not crystallize during the cooling/reheating cycle mentioned above.

**Figure 2:**
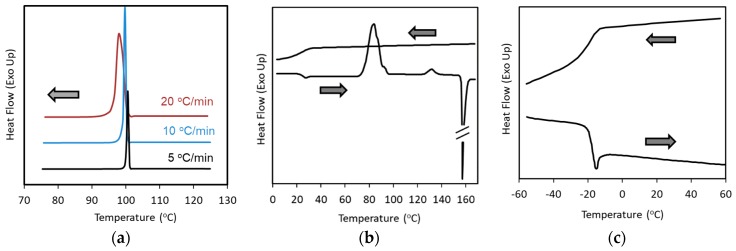
Examples of cooling/reheating differential scanning calorimetry (DSC) curves from the melt: (**a**) cooling curves of haloperidol (Class 1) at various cooling rates, as indicated in the figure; (**b**) cooling/reheating curves of acetaminophen (Class 2); and (**c**) cooling/reheating curves of fenofibrate (Class 3).

Examples are shown in Figure 2. Haloperidol, a Class 1 compound, always crystallizes at 100 °C during cooling from the melt, regardless of the cooling rate achievable by conventional DSC (Figure 2a) [33], which means that crystallization is entirely governed by the temperature. It should be noted that the crystallization temperature of some Class 1 compounds such as tolbutamide depends on the cooling rate [33]. Class 1 compounds can be further divided into two groups according to their crystallization behavior during cooling in liquid nitrogen, whereby compounds that crystallize and remain amorphous are categorized as Class 1a and Class 1b, respectively [34]. This difference is likely to be analogous to the dependence of the crystallization temperature on the cooling rate mentioned above, that is, haloperidol and tolbutamide can be identified as Class 1a and Class 1b compounds, respectively. In the case of haloperidol, crystallization is inhibited when the melt is cooled at a rate faster than 100 °C/s to produce a mesophase [33]. Acetaminophen, a Class 2 compound, does not crystallize during cooling, but crystallizes during the subsequent reheating (Figure 2b). Fenofibrate, a Class 3 compound, does not crystallize during the cooling/reheating cycle (Figure 2c). Table 2, Table 3 and Table 4 summarizes examples of compounds belonging to each class.

Average parameters are also presented in the table for each class of compounds. The molecular weight shows an increase with increasing classification number, which reflects the importance of the complexity of the molecular structure. The melting enthalpy also increases with increasing classification number, which can be explained in terms of the strength of the molecular interactions. On the other hand, the effect of the melting temperature was opposite to the expectation, while the effect of the fragility was not clear. However, the effect of the fragility is difficult to evaluate, because this parameter could not be calculated for most Class 1 compounds. Moreover, the fragility obtained for chlorpropamide exhibited an unusual value, 219, which significantly influenced the overall average.

**Figure 3:**
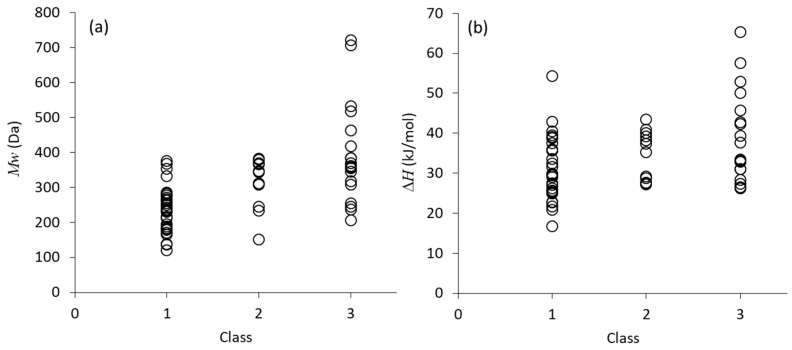
Visualization of: (**a**) molecular weight; and (**b**) melting enthalpy of compounds belonging to each class.

Figure 3 visualizes individual data of molecular weight and melting enthalpy of compounds in each class. Figure 3a clearly shows that all compounds with the molecular weight larger than 400 Da are involved in Class 3, whereas the molecules smaller than 200 Da are not included in Class 3 at all. However, molecular weight was found to be the only parameter that shows some extent of correlation with the crystallization tendency, if all the data are plotted, as presented in Figure 3. As an example, Figure 3b shows relationship between the melting enthalpy and crystallization tendency. Although the averaged values indicated correlation with the crystallization tendency, it is not obviously statistically meaningful. Other structural/thermodynamic parameters did not exhibit any correlations with the crystallization tendency, either. Special attention to molecular weight was also made by Mahlin et al. [24], who found the molecules larger than 300 Da to be good glass formers during formulation processes. Note that the structural feature of compounds that may be correlated with the crystallization tendency, as shown in Table 1, has been mainly concluded by observing series of compounds that have similarity in their chemical structure. When variety of compounds is collected for examination, focus on single parameter does not seem to be sufficient. The combination of molecular volume and melting enthalpy was reported to be an excellent predictor of the crystallization tendency by Wyttenbach et al., based on theoretical considerations centered on the so-called Prigogine–Defay ratio [35]. In their study, the trend of the *T_g_*/*T_m_* ratio also agreed with the expected trend; interestingly, the *T_g_*/*T_m_* parameter was also shown to be correlated with the Prigogine–Defay ratio [35,41].

A common strategy to improve biopharmaceutical performance of poorly soluble candidates includes increase in hydrophilicity, which frequently has trade-off relationship with affinity to therapeutic targets. However, another approach may be suppression of crystallization tendency based on the information described in Table 1 to increase applicability of ASD. As noted below, suppression of crystallization tendency may also be related to increase in supersaturation ability after dissolution. Further understanding on relationship between chemical structure and crystallization tendency should increase options of chemical modification strategy of candidate compounds.

Alternatively, the critical cooling rate for achieving vitrification has also been employed for the classification; for example, compounds that crystallize even at 750 °C/min were classified as Class 1, those with moderate crystallization ability and that can be vitrified at ca. 10–20 °C/min were designated as Class 2, while Class 3 compounds only require a very slow cooling rate, below 2 °C/min, for vitrification [38,42]. Despite the different criteria employed, the classifications based on this methodology agreed well with those in Table 2, Table 3 and Table 4, except that tolbutamide and cinnarizine were placed in Classes 2 and 3, respectively [38].

**Figure 4:**
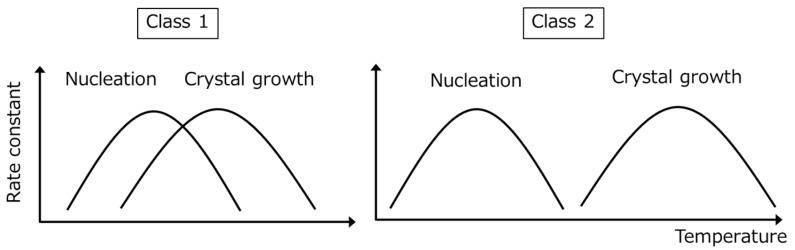
Schematic representation of the temperature dependence of the nucleation and crystal growth temperatures for Classes 1 and 2 compounds.

The different behavior of Classes 1 and 2 compounds likely reflects differences in nucleation and crystal growth temperatures (Figure 4). For Class 1 compounds, the optimum nucleation and crystal growth temperatures should be close to each other; hence, after reaching an optimum temperature where both nucleation and crystal growth proceed, the melt can crystallize. This process is expected to be based on homogeneous nucleation. In contrast, the optimum nucleation temperature for Class 2 compounds should be located far below the optimum crystal growth temperature. Thus, the melt must be first cooled to the nucleation temperature range and then heated to the crystal growth temperature for crystallization to proceed. However, if the cooling rate is sufficiently slow, there is a finite chance for nucleation to occur at the optimum crystal growth temperature even though the nucleation rate is very low, which could explain the similar classifications produced by the two methods.

**Figure 5:**
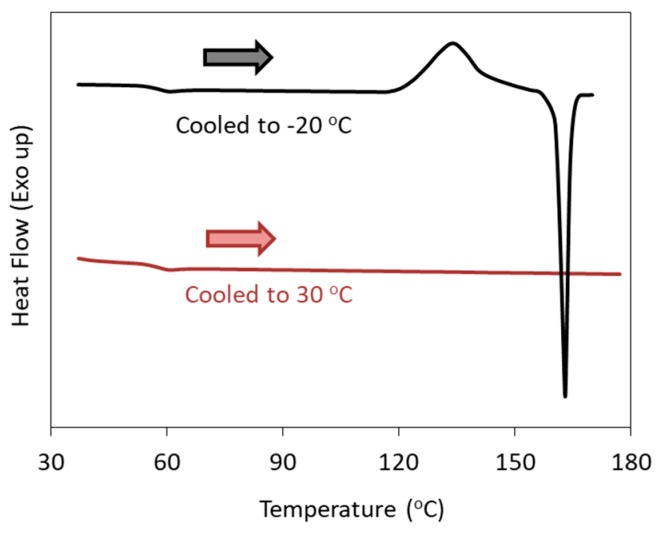
Reheating DSC curves of celecoxib melt, illustrating the dependence of the cold crystallization on the target temperature of the cooling process (shown in the figure).

Figure 5 shows reheating DSC curves of celecoxib melt, illustrating the dependence of the cold crystallization on the target temperature of the cooling process [43]. When the melt was cooled down to −20 °C, a crystallization exotherm was observed during the subsequent heating process. However, no crystallization was observed when the melt was cooled down to 30 °C, although celecoxib is known as a Class 2 compound. Our investigation revealed that the optimum nucleation temperature of celecoxib was ca. −50 °C; thus, cooling to 30 °C was obviously not enough for inducing nucleation. In the classification criteria discussed above, the minimum temperature of the cooling process is not specified. However, a poor understanding of the nucleation process may result in the misclassification of a particular compound.

The different behavior of Classes 2 and 3 compounds is likely due to the different strength of their molecular interactions. Thus, the presence of neighboring molecules during the crystallization cannot be ignored, and the crystallization is based on heterogeneous nucleation.

## 3. Relationship between Crystallization Tendency and Isothermal Crystallization

**Figure 6:**
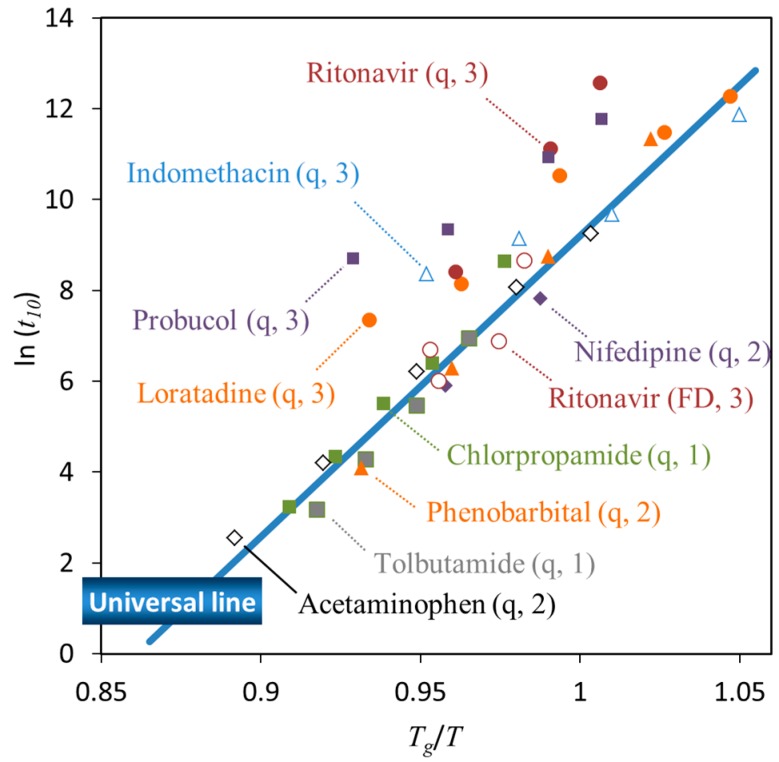
Initiation time of crystallization (*t*_10_, min) as a function of *T_g_*/*T*. The q and FD labels in the parentheses indicate that the glass was prepared by quenching and freeze-drying, respectively. The numbers in parentheses denote the crystallization tendency classification. The universal line is the best fit for Classes 1 and 2 compounds (ln(*t*_10_) = 66.2*T_g_*/*T* − 57.0).

The crystallization tendency discussed above does not directly correlate with the physical stability under isothermal conditions. However, these two processes do have some indirect relationships. Figure 6 shows the time to reach 10% crystallinity (*t*_10_, expressed in minutes) for pharmaceutical glasses as a function of *T_g_*/*T*, where *T* is the storage temperature [44]. These data were acquired for quenched glass pellets under dry conditions. Crystallization has frequently been observed to start at the surface [45,46]. Since the pellets have a very small surface area, the surface effects on the crystallization were almost eliminated in this experiment. Clearly, the data corresponding to most compounds fell on a universal line; in particular, the compounds located on the line belonged to Classes 1 and 2. The other compounds, which exhibited better stability especially above *T_g_*, belonged to Class 3.

The above data were obtained by fitting the crystallinity value at each time point to the Avrami–Erofeev equation. The obtained Avrami exponents are shown in Table 5 Smaller Avrami exponents were obtained for higher classification numbers, which indicates that the nucleation mechanism becomes more homogeneous with decreasing classification number. This hypothesis is also supported by a previous in-situ analysis of the isothermal crystallization process of tolbutamide and acetaminophen using synchrotron X-ray diffraction [44].

The crystallization of some glasses was observed to start at the surface. In the case of indomethacin, crystallization is enhanced with decreasing particle size, which is most likely due to the increasing surface area [45]. Moreover, the crystallization of indomethacin glass particles is retarded by a polymer coating of the surface [46]. Quenched ritonavir glass exhibited higher stability relative to that of the compounds located on the universal line in Figure 6. However, the stability of freeze-dried ritonavir glass could be explained by the universal line, which is likely due to the increase in surface area [47]. The lower packing of the glass structure might also partially contribute to eliminate the effect of molecular interactions. The surface effects are usually explained in terms of the higher mobility of surface molecules [48], due to a decreased number of nearest neighbor molecules [49].

The results in Figure 6 suggest that the physical stability of Classes 1 and 2 compounds was strongly affected by the temperature. In these cases, physical stabilization of the glasses appears difficult to achieve without adding excipients. However, as the crystallization of Class 3 compounds is influenced by molecular interactions, physical stabilization of these systems may be achieved by manipulating these interactions. In fact, quenched ritonavir glass had higher stability compared to that of the freeze-dried glass, as discussed above.

Sub-*T_g_* annealing based on this strategy was found to be an effective strategy for stabilizing ritonavir glass [50]. For example, ritonavir glass annealed at 40 °C for two days was much more stable compared to fresh glass. The fresh glass reached a crystallinity of 58% after annealing at 60 °C for six days, whereas the glass pre-annealed at 40 °C reached a crystallinity of only 8% after the same annealing procedure at 60 °C. Structural analysis revealed a change in the packing volume and hydrogen-bonding pattern during the pre-annealing at 40 °C, which was the most likely source of the stabilization. Such pre-annealing strategy did not work for Classes 1 and 2 compounds [50].

## 4. Non-Ideal Crystallization of Practical Glasses

The discussion presented above is based on observation under well-defined conditions, where effect of mechanical stress, moisture sorption, and surface area were minimized. Crystallization behavior of practical glasses, especially in the case of powder samples, may not be explained in such an ideal manner. Glasses prepared by grinding typically exhibit lower stability than the intact ones most likely because of remaining nuclei and/or small crystals that cannot be detected by X-ray powder diffraction. In the observation of Crowley et al. [51], crystallization behavior of indomethacin glasses prepared by cryogenic grinding of various crystal forms depended on the initial crystal form used, suggesting that the ground glasses remembered their original forms even after the grinding. In their study, they also observed significant differences in the crystallization rates of ground and quenched glasses. Thus, although grinding is a simple process to prepare amorphous form in a laboratory scale, it is not recommended because of difficulty in transformation into the amorphous state in a molecular level.

**Figure 7:**
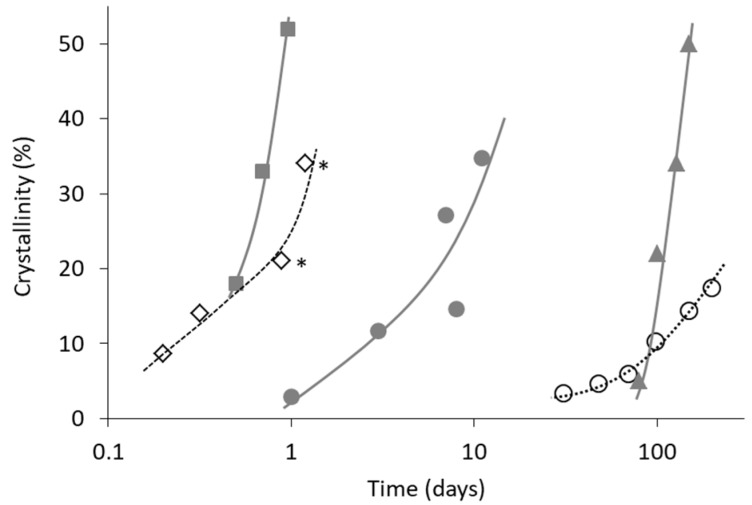
Isothermal crystallization of indomethacin glasses at 30 °C under dried condition. (■) Quenched and ground for 6 min. Crystallized to form γ [51]. (◇) Quenched and ground. Crystallized to form α except that symbols with asterisk involves small amount of form γ [54]. (●) Quenched and cryoground. Crystallized to mixture of form α and γ (our data). (▲) Quenched. Crystallized to form γ [55]. (○) Quenched and stored in DSC pan (our data). Crystallized to form α which contains small amount of form γ.

Even for melt–quenched glasses, application of subsequent grinding process can accelerate crystallization [52]. Moreover, very weak stresses such as crack formation [53] and transfer to different vessels [52] are also suspected as causes of nucleation. Figure 7 shows comparison of crystallization behavior of melt–quenched indomethacin glasses at 30 °C with or without grinding process before the storage. In the absence of the grinding process, the quenched glass remained completely in an amorphous state for more than one month. However, if the grinding process is applied for the melt–quenched glass, crystallization is initiated within one day. This comparison clearly indicates significant effect of the grinding process on the crystallization behavior, which appeared to be due to increase in the surface area and mechanical stress. It is also interesting to note that the crystal form obtained was not identical in these examples. Since no relevance between the preparation process and crystal form could be found, it might be because of difference in impurity profiles.

Crystallization of nifedipine is very sensitive to various factors including moisture sorption and mechanical stress. Thus, extensive care is required to investigate the ideal crystallization behavior as presented in Figure 6. In our experiments, crystalline powder was dried in a vacuum oven at 50 °C and stored in a desiccator with silica gel before use. Then, the dried powder was loaded in a hermetically sealed pan under flow of dried nitrogen air, and subjected to the melt–quench procedure to initiate the stability study. Only after such careful treatment, the data which could be explained by the universal line were obtained.

**Figure 8:**
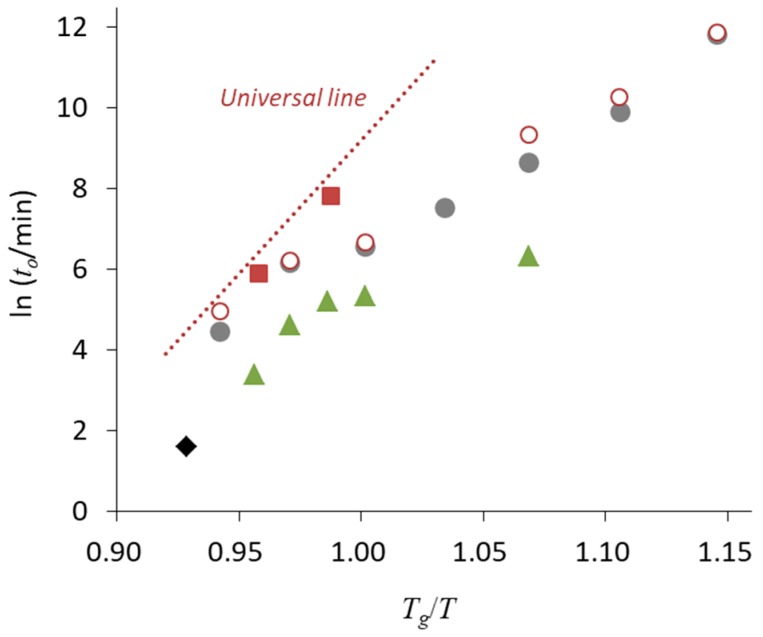
Onset crystallization time (*t*_o_, min) of nifedipine glass as a function of *T_g_*/*T*. (■) After the pretreatment (see text), quenched in hermetically sealed pan (our data) [44]. (○) Quenched in sealed DSC pan without pretreatment (our data). (●) Quenched in DSC pan [57]. (▲) Quenched on glass slides and crystallization was observed by polarized light microscopy [56]. Cracked glasses were excluded from the analysis. (◆) Quenched in DSC pan [58]. All the literature data were recalculated using the *T_g_* value of 45.5 °C. Definition of onset crystallization time, which is analogous to *t*_10_, is slightly different depending on literature, but its impact is ignorable in the analysis here.

Therefore, the data for nifedipine crystallization found in literature are usually faster than the expectation from the universal line. Figure 8 shows onset crystallization time of nifedipine glasses extracted from various literature sources. As already presented in Figure 6, the nifedipine data obtained after the careful treatment mentioned above were explainable by the universal line. However, the crystallization was much faster for the glasses loaded in normal sealed pans without pretreatment. Observation using polarized light microscopy by Bhugra et al. was done in a very careful manner [56], where cracked glasses were eliminated from the analysis, because it can enhance the crystallization. However, the crystallization was much faster, presumably because the glasses could not be shielded from outer atmosphere completely.

**Figure 9:**
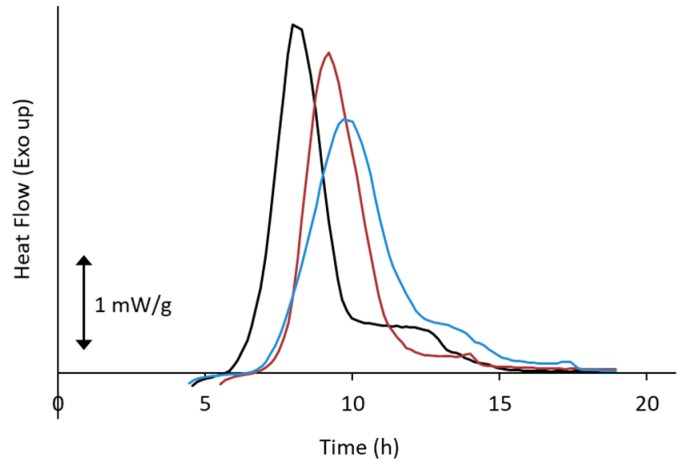
Effect of compression pressure on crystallization heat flow curves of sucrose glasses investigated by isothermal microcalorimetry (30 °C). Freeze-dried sucrose was compressed at pressure of ca. 2.5 MPa (black), 0.5 MPa (red), or 0.1 MPa (blue) for 10 s and subjected to the measurement.

Compression process is also recognized to affect the crystallization kinetics. Figure 9 shows effect of compression pressure on crystallization of sucrose glass investigated by isothermal microcalorimetry [59]. Initiation time for crystallization was rarely influenced below 0.5 MPa; however, crystal growth was enhanced with increasing pressure. It was shortened at 2.5 MPa, suggesting that condensation of glass structure can enhance nucleation after application of such relatively weak compression force. Similarly, Ayenew et al. reported that cold crystallization of indomethacin glass was enhanced by compression at ca. 43.7 MPa [60]. In their study, uncompressed glass, which was prepared by cooling the melt at 0.2 °C/min, was observed to crystallize at 121.4 °C during subsequent reheating at 5 °C/min. However, it decreased to 114.1, 113.1, and 112.7 °C, if the compression was applied for 1 s, 2.5 min, or 5 min, respectively. Rams-Baron et al. observed that isothermal crystallization of etoricoxib was significantly enhanced after compression at 300 MPa; however, it could be prevented by mixing with polyvinylpyrroridone (PVP), where investigations were done under the identical relaxation time conditions [61]. This result indicated that physical barrier by excipients were very effective for inhibiting pressure-induced nucleation.

Based on the universal line, the only requirement for assuring three-year stability of pharmaceutical glasses at 25 °C is the *T_g_* higher than 48 °C [44]. Its applicability to practical glasses which are produced under various mechanical stresses without protection from outer atmosphere is discussed next.

## 5. Relevance to Formulation Research

Practical ASDs cannot be manufactured by the melt–quenching procedure. The crystallization tendency during practical formulation processes is also of great interest to formulators. This is similar but different phenomena from the crystallization from the melt; therefore, some attempts have been made to find relevance between them. The tendency to crystallize from solution after drying is of great importance for evaluating applicability of spray-drying. Eerdenbrugh et al. investigated the crystallization of 51 compounds after removal of the solvent by spin coating, in order to identify possible correlations between the crystallization tendencies evaluated by thermal analysis upon cooling/reheating and during drying from solutions [62]. In their analysis, the compounds that exhibited a tendency to crystallize immediately after spin coating were denoted as Class 1, those that crystallized within one week were categorized as Class 2, and the remaining compounds were regarded as Class 3. Approximately 76% of the compounds classified as Class 1 by DSC were also assigned to Class 1 by the spin coating method, whereas 76% of the compounds classified as Class 3 by DSC were again classified in the same group by the spin coating approach. The *T_g_* values seemed to break the correlation between the two classification methods.

The relevance to vitrification during milling has also been studied. Blaabjerg et al. reported minimum milling times to achieve vitrification as 90 and 270 min for Classes 3 and 2 compounds, respectively, whereas no vitrification was achieved for any of the Class 1 compounds [42]. It should be noted that some Classes 2 and 3 compounds failed to form amorphous systems, most likely due to their low *T_g_*. This observation suggests that the crystallization tendency from the melt can be used to guide the design of hot-melt extrusion processes, along with additional information on the *T_g_* values.

Thus, applicability of ASD technology to poorly soluble candidates may be judged from the crystallization tendency determined by DSC with the information on *T_g_*. In the formulations, polymeric excipients are used for two purposes: physical stabilization and improvement of dissolution/supersaturation behaviors [12]. Class 3 compounds can be expected to be transformed into the amorphous state using typical formulation processes for ASDs even without excipients. Main purposes of addition of polymeric excipients in the ASD design are to raise *T_g_* for ensuring storage stability and to improve dissolution behavior. Amount of excipient may be kept small for these compounds. Even if amount of the drug exceeds solid solubility limit, the drug is expected to remain in the amorphous state [63]. In contrast, Class 1 compounds must be completely mixed with excipients in a molecular level for the successful transformation to the amorphous state. The amounts of excipients are expected to be larger compared to that for Class 3 compounds. Typically, solid solubility of drug in polymeric matrix under ambient temperature is below 30%, sometimes below 10%, depending on combination of drug and excipient [21,22,23]. Moreover, it is frequently observed that the effective polymer for physical stabilization and dissolution improvement is different. Hydroxypropyl methylcellulose acetate succinate frequently offers great effect for maintaining high level of supersaturation; however, its miscibility with drug is typically low. In contrast, PVP and its derivatives have relatively high miscibility with drug, but its supersaturation effect cannot be maintained for long duration in many cases. It must be recognized as well that prepared ASDs are not necessarily in the equilibrium state. If ASDs are prepared under an elevated temperature condition, as in case of hot-melt extrusion, the mixing state at this temperature may be kinetically frozen even after cooling to ambient temperature. In spray-drying, the drug and excipient molecules may be separated based on the difference in their molecular weights, because diffusion rate during evaporation process is different [64,65]. This kinetically-separated structure may also be frozen after the drying [65,66]. If solvents are used during the preparation, as in the cases of spray-drying and coprecipitation, the mixing state of the ASDs is affected by the solvent species [67]. In such cases, the mixing state may change with time [23].

**Figure 10:**
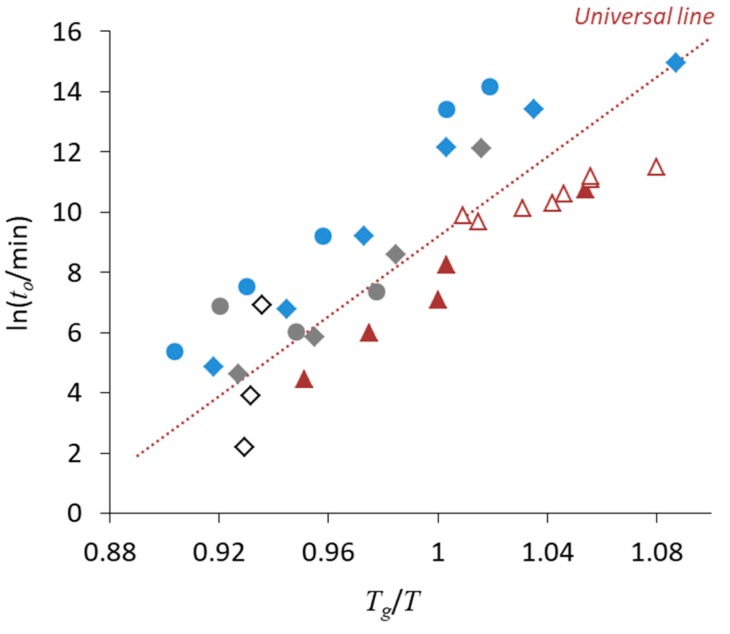
Onset crystallization time (*t*_o_, min) of various ASDs as a function of *T_g_*/*T*. (◆,◆,◇) Nifedipine/PVP ASDs prepared by melt–quench [68,69] followed by milling [58], respectively. (●, ●) Phenobarbital/PVP ASDs prepared by melt–quench [68,69]. (▲,△) Sanofi–Aventis compound/HPMCP ASDs prepared by spray-drying stored under dried and humid conditions, respectively [70]. Definition of onset crystallization time, which is analogous to *t*_10_, is slightly different depending on the study, but its impact is ignorable in the analysis here. HPMCP, Hydroxypropyl methylcellulose phthalate.

How the universal line in Figure 6 is applicable to multi-component ASDs is of great interest. Figure 10 shows comparison of the onset crystallization time of single phase ASDs appearing in the literature. As an overall trend, the universal line seems to work even for the multicomponent systems. Comparison of nifedipine/PVP ASDs from three different stuides implies that milling enhances the nucleation. However, presence of polymeric excipients appears to stabilize the ASDs more than expected from change in *T_g_* (i.e., molecular mobility), most likely because of dilution effect and interaction with drug. The result for Sanofi–Aventis compounds is the most informative from a practical point of view, because the ASD was prepared by spray-drying. Stability of this ASD is a little lower but roughly agrees with the universal line regardless of absence/presence of the moisture. When each dataset is fitted with a regression line, their slopes are almost the same, suggesting that activation energy for nucleation does not significantly depend on the type of ASDs. Design of accelerated physical stability test may be possible for ASDs based on this information.

## 6. Relevance to the Dissolution Benefits of ASDs

The greatest advantage of ASDs is that they can achieve supersaturation of poorly soluble drugs. Supersaturation can be maintained unless crystallization occurs in aqueous environments [71]. Thus, it is important to understand the controlling factors that cause crystallization of pharmaceutical glasses in aqueous media. Our preliminary investigation revealed that crystallization proceeds immediately above *T_g_* of the solid [33]. Blaabjerg et al. reported that the degree of supersaturation tended to be high for good glass formers, but no correlation was found between crystallization tendency and supersaturation lifetime [72]. We have shown that amount of orally absorbed fenofibrate was correlated with liquid–liquid phase separation concentration, which is analogous to degree of supersaturation, if the oral absorption was limited by solubility [13]. Thus, suppression of the crystallization tendency can be an option of chemical modification of the poorly soluble candidates instead of increasing aqueous solubility. Alhalaweh analyzed the relationship between the crystallization tendencies from the melt and those in solution, highlighting the need to consider structural factors to improve the correlation between these tendencies [73]. From the viewpoints of physical stability and supersaturation ability, Class 3 compounds seem to be suitable candidates for ASDs. In fact, most of the marketed ASDs consist of Class 3 compounds [74].

## 7. Summary

This review provides the classification of the crystallization tendencies of pharmaceutical compounds, focusing on its relevance for the glass properties. Possible relationships discussed in this review are summarized in Table 6. In addition to its effectiveness for describing the physical stabilities of ASDs, this classification provides important insights into the glass properties. The investigation of the crystallization mechanism of small organic compounds is an attractive subject because of their structural diversity and complicated molecular interactions, in contrast to the inorganic compounds that have dominated the field of glass science so far. Further progress in this field can make a significant contribution to both basic glass science and practical developmental studies of pharmaceutical products.

Preparation of practical formulations involves some procedure to enhance nucleation. Ideality of the nucleation/crystallization behavior is destroyed by application of some activation processes such as milling. However, presence of polymeric excipients can contribute to stabilization, presumably due to dilution effect and its interaction with drug molecules. As a result, deviation from the ideal behavior due to formulation processes is suppressed for enabling rough prediction of the crystallization time. Design of accelerated physical stability test may be possible for ASDs based on this observation.

Although compounds in any classes can be formulated as ASDs, Class 3 compounds obviously have the highest applicability. In addition to their high physical stability, they may have an advantage in supersaturation ability that has great contribution to enhanced absorption. Therefore, chemical modification to decrease crystallization tendency may be considered as an option for drug design instead of increasing solubility.

## Figures and Tables

**Table 1:** Features of good glass formers based on small organic molecules.

Chemical-Structural Features	Physicochemical Features
Large molecular weight	Large melting enthalpy/entropy
Low number of benzene rings	High melting temperature
Low symmetry	Large crystal/amorphous energy difference
Large number of rotatable bonds	Large fragility
High branching degree	Large *T_g_*/*T_m_*
Large number of electronegative atoms	Large viscosity above *T_g_*

*T_g_*, glass transition temperature; *T_m_*, melting temperature.

**Table 2:** Examples of Class 1 compounds.

Compounds	*M*_w_ (Da)	*T_m_* (°C)	*T_g_* (°C)	*T_g_*/*T_m_*	Δ*H* (kJ/mol)	*m*	Reference
Antipyrin	188	111	−25	0.65	25.2	81	[31]
Anthranilic acid	137	147	5	0.66	22.8	-	[26]
Atenolol	266	153	22	0.69	37.5	-	[26]
Atovaquone	367	219	-	-	33.5	-	[35]
Benzamide	121	127	−10	0.66	21.7	-	[26]
Benzocaine	165	89	−31	0.67	22.6	-	[26]
Caffeine	194	237	72	0.68	20.8	-	[26]
Carbamazepine	236	192	61	0.72	25.5	-	[26]
Chlorpropamide	277	118	17	0.74	27.4	219	[31]
Chlorzoxazone	170	191	38	0.67	25.6	-	[26]
Clofibric acid	215	121	-	-	29.0	-	[35]
Diflunisal	250	213	-	-	35.6	-	[35]
Felbinac	212	164	24	0.68	29.8	-	[26]
Flufenamic acid	281	135	17	0.71	27.1	78	[26,36]
Griseofulvin	353	218	89	0.74	39.1	74	[26,37]
Haloperidol	376	152	33	0.72	54.3	-	[26]
Indoprofen	281	212	50	0.67	36.0	-	[26]
Lidocaine	234	68	−39	0.69	16.7	-	[26]
Mefenamic acid	241	231	-	-	39.4	-	[35]
Naproxen	230	157	56	0.77	32.4	-	[35,38]
Nepafenac	254	183	-	-	42.8	-	[35]
Phenacetin	179	136	2	0.67	31.5	-	[26]
Piroxicam	331	201	-	-	35.6	-	[35]
Probenecid	285	199	-	-	40.4	-	[35]
Saccharin	183	228	-	-	29.5	-	[35]
Salicylic acid	138	159	-	-	24.9	-	[35]
Theophylline	180	272	94	0.67	29.6	-	[26]
Tolbutamide	270	128	5	0.69	26.2	122	[31]
Tolfenamic acid	262	213	63	0.69	38.8	-	[26]
Average	237	172	27	0.69	31.1	115	-

*M*_w_, molecular weight; Δ*H*, melting enthalpy; *m*, fragility. Although the fragility can be determined by various methods, the evaluation based on the temperature dependence of *T_g_* is preferentially employed because it exhibits the best correlation with the crystallization tendency [31].

**Table 3:** Examples of Class 2 compounds.

Compounds	*M*_w_ (Da)	*T_m_* (°C)	*T_g_* (°C)	*T_g_*/*T_m_*	Δ*H* (kJ/mol)	*m*	Reference
Acetaminophen	151	169	23	0.67	27.2	77	[31]
Bifonazole	310	149	16	0.68	39.2	76	[31]
Celecoxib	381	163	58	0.76	37.4	85	[26]
Cinnarizine	369	120	7	0.71	40.9	84	[31]
Clofoctol	365	88	−4	0.75	35.2	70	[26]
Dibucaine	343	65	−35	0.70	29.2	132	[26]
Droperidol	379	143	29	0.73	40.0	108	[26]
Flurbiprofen	244	115	−5	0.69	27.4	88	[31]
Nifedipine	346	172	46	0.72	38.2	112	[31]
Phenobarbital	233	174	42	0.70	28.7	96	[31]
Phenylbutazone	308	106	−6	0.70	27.6	79	[36]
Tolazamide	311	172	18	0.65	43.4	18	[26]
Average	312	136	16	0.71	34.5	85	-

**Table 4:** Examples of Class 3 compounds.

Compounds	*M*_w_ (Da)	*T_m_* (°C)	*T_g_* (°C)	*T_g_*/*T_m_*	Δ*H* (kJ/mol)	*m*	Reference
Aceclofenac	354	153	10	0.66	42.3	25	[26]
Clotrimazole	345	141	28	0.73	33.3	63	[31]
Curcumin	368	182	62	0.74	50.1	87	-
Felodipine	384	147	45	0.76	31.0	66	[26]
Fenofibrate	361	80	−19	0.72	33.0	82	[31]
Ibuprofen	206	76	−44	0.66	26.5	75	[31]
Indomethacin	358	161	45	0.73	37.6	85	[31]
Itraconazole	706	168	58	0.75	57.6	731	[26]
Ketoconazole	531	147	44	0.75	52.9	97	[31]
Ketoprofen	254	95	−3	0.73	28.3	67	[31]
Loratadine	383	134	35	0.76	27.3	72	[31]
Miconazole	417	86	1	0.76	32.8	61	[26]
Nilutamide	317	155	33	0.72	31.0	106	[26]
Nimesulide	308	150	21	0.70	33.4	103	[26]
Pimozide	462	219	54	0.66	42.7	170	[26]
Probucol	517	126	27	0.75	39.3	138	[39]
Procaine	236	61	−39	0.70	26.2	90	[31]
Ribavirin	244	168	56	0.75	45.7	70	[40]
Ritonavir	721	122	47	0.81	65.3	86	[31]
Average	393	135	24	0.73	38.8	120	-

**Table 5:** Ranges of Avrami exponents for isothermal crystallization.

Classification	Compound	Avrami Exponent
Class 1	Tolbutamide	3.7–4.6
	Chlorpropamide	3.0–4.2
Class 2	Acetaminophen	2.1–3.0
	Nifedipine	2.0
Class 3	Ritonavir	2.2–3.1
	Indomethacin	1.0–2.6
	Loratadine	1.1–1.5
	Probucol	1.2–1.3

**Table 6:** Relevance of crystallization tendency classification for glass properties.

*With increasing classification number:*
• Nucleation becomes more heterogeneous
• The nucleation barrier becomes larger
• Surface effects become more important
• Vitrification during the formulation processes may become easier
• The supersaturation ability may increase
• The universal line in Figure 6 is applicable for Class 1 and 2 compounds, whereas the stability of Class 3 compounds may be better
• Stabilization may be achieved via thermal treatment
